# ProcessOptimizer, an Open-Source Python Package for Easy Optimization of Real-World Processes Using Bayesian Optimization: Showcase of Features and Example of Use

**DOI:** 10.1021/acs.jcim.4c02240

**Published:** 2025-02-07

**Authors:** Søren Bertelsen, Sigurd Carlsen, Søren Furbo, Morten Bormann Nielsen, Aksel Obdrup, Rolf Taaning

**Affiliations:** † Department of Automation and Process Optimisation, Digital Science and Innovation, Novo Nordisk A/S, 2760 Måløv, Denmark; ‡ Danish Technological Institute, Kongsvang Allé 29, 8000 Aarhus C, Denmark; § Department of Digitalization, Global IT & Digital, Topsoe A/S, 2800 Kongens Lyngby, Denmark; ∥ Department of Late-Stage Chemical Development, Chemistry Manufacture and Control, Novo Nordisk A/S, 2880 Bagsværd, Denmark; ⊥ Teal Medical, 2300 Copenhagen, Denmark

## Abstract

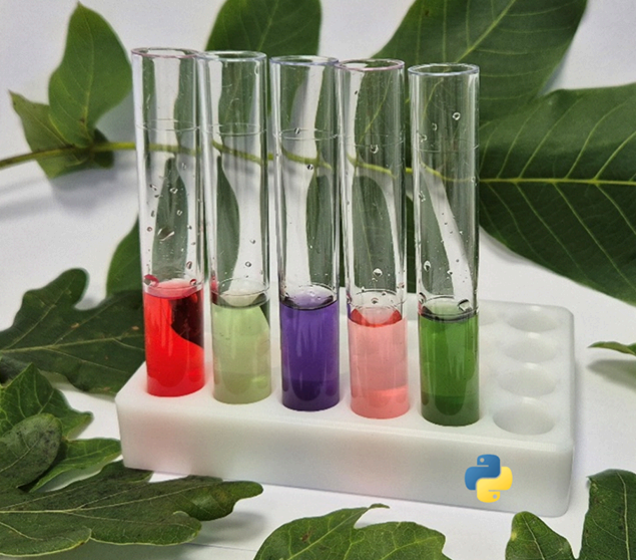

ProcessOptimizer is a Python package designed to provide easy access to advanced machine learning techniques, specifically Bayesian optimization using, e.g., Gaussian processes. Aimed at experimentalist scientists and applicable to process and product optimizations in various fields, this package simplifies the optimization process, offering features such as benchmarking, noise addition/removal, multiobjective optimization, batch-mode operation, and comprehensive plotting features. The present publication focuses on ease of use by presenting an optimization of a chemical reaction to produce a specific color, such as leaf green.

## Introduction

Process optimization is a cornerstone of scientific research and industrial applications. It is the strategic approach of fine-tuning various parameters to achieve the best possible outcome in terms of efficiency, yield, or cost-effectiveness. Proper application of structured process optimization often produces significant cost savings, improved product quality, enhanced performance, and increased productivity. In the context of experimental sciences, process optimization can help identify critical control parameters, find and apply optimal conditions, and ensure highly reliable regression models.^1^

**Figure 1:**
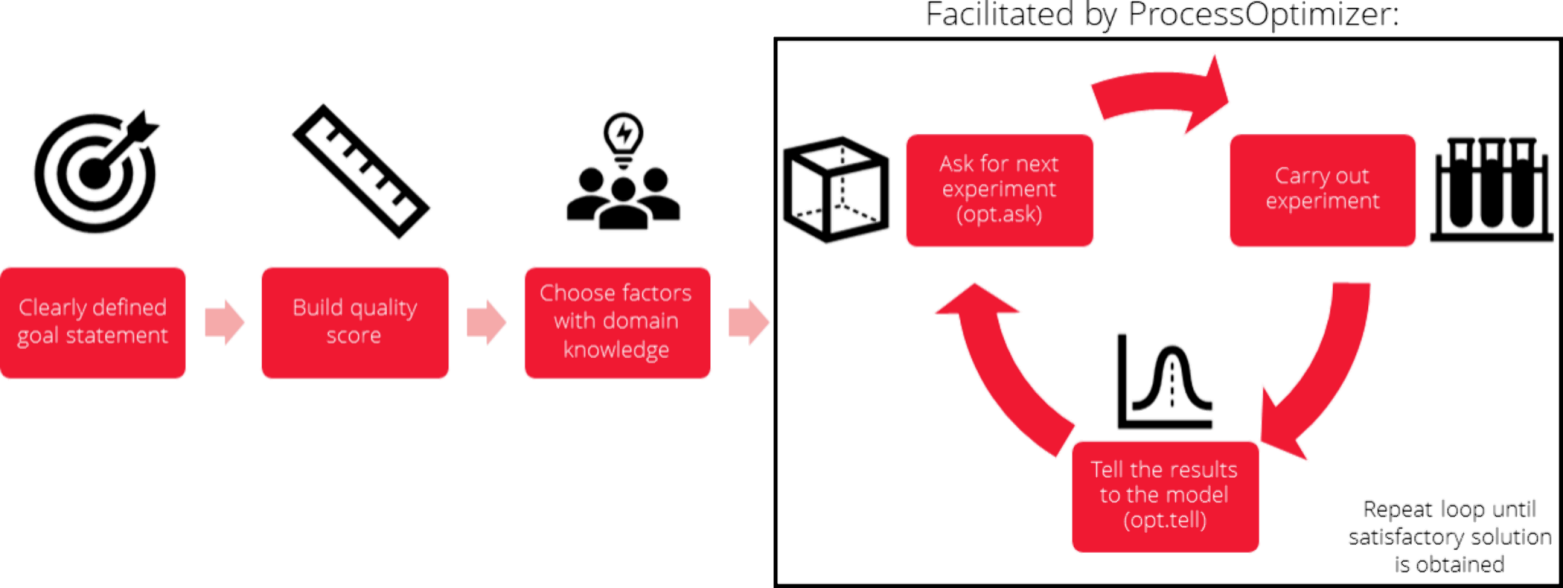
Overall workflow of Bayesian optimization. The initial steps are common to many optimization strategies, and ProcessOptimizer is built to make the iterative optimization loop as simple to execute as possible.

Optimizing a process involves multiple stages, as illustrated in Figure 1. The user first specifies optimization goals depending on the problem at hand, after which one must choose an appropriate metric to measure progress toward these goals, the factors to vary, and what values to allow for these factors. All of these steps require significant domain knowledge to ensure a successful process optimization. Once these decisions have been made, the process that is normally described as process optimization can begin.

The intuitive approach to process optimization is often a simple trial-and-error method, where changes are made randomly or one factor at a time and outcomes are observed. This approach, however, is time-consuming and lacks precision. The need for a more systematic and efficient approach led to the development of “Design of Experiments” (DoE).^2^

DoE emerged as a structured, organized method to study the effects of various factors on a process. Early developments within DoE focused on screening designs, which aimed to identify the most influential factors among many. This was particularly useful for scientists who needed to determine the key control parameters in complex processes.^3^

Modern optimal designs were later introduced to determine the best combination of factor levels to estimate the model parameters. This approach allowed for more efficient exploration of the experimental space, easier handling of constraints, and more complex models.^4^

One of the most significant advancements in modern DoE is Bayesian optimization, a sequential design strategy for global optimization of black-box functions.^5^ Bayesian optimization considers the past evaluation results to choose the next input, allowing for more efficient exploration and exploitation of the process. This technique is particularly useful for scientists aiming to ensure either as large incremental improvements toward the objective(s) as possible (exploitation) or as large as possible information gain at each iteration (exploration). The iterative nature of the continued learning gives specific advantages, as it allows every single experiment design to be informed by the entire existing body of data. This is in opposition to classical DoE, where the majority of the runs are chosen at the time where the least amount of data is available.

Today, Bayesian optimization is being widely used in various fields, from hyperparameter tuning in machine learning models to process optimization in the experimental sciences. The current rise of self-driving laboratories often relies on Bayesian optimization.^6^ Many software packages for Bayesian optimization exist, with various degrees of user friendliness as well as a number of features presented to the user. Comparison through benchmarking is difficult because many packages are highly customized to specific fields of intended use and outcomes will vary depending on the objective of the optimization, as in the classical explore/exploit trade-off. To facilitate a qualitative comparison between the state-of-the-art packages within Bayesian optimization for life sciences, a noncomprehensive list of Python packages is presented in Supporting Information S1.

However, the application of Bayesian optimization often requires a high level of expertise in coding and machine learning, which can be a barrier for many scientists. Here we introduce an open-source Python package that simplifies the setup of Bayesian optimization for real-world processes, making it more accessible to researchers and practitioners in various fields.^7^ ProcessOptimizer is a further development from another excellent open-source Python package, Scikit-Optimize.^8^ Numerous new features have been added, and many default settings have been set to reflect ProcessOptimizer’s intended use in optimizing real-world processes, as opposed to machine learning problems.

ProcessOptimizer is a Python package that brings the power of Bayesian optimization to the fingertips of experimental scientists and process optimization professionals working on real-world (physical) processes. It is designed with simplicity and ease of use in mind, allowing users to leverage advanced machine learning models without requiring extensive coding or machine learning expertise.

## Example of Use

In this paper, we illustrate the ease of setup and use of ProcessOptimizer, with a practical example from the field of chemistry: the goal is to produce 240 μL of a liquid having a color as close to a chosen color as possible (e.g., leaf green). This is a far simpler system than what would normally be the object of a real-world optimization but serves for illustrative purposes.

The objective of this specific optimization is to minimize the Δ*E* value in the L*a*b* color space.^9^ As factors, our chemist chooses (1) an amount of a universal pH indicator between 5 and 40 μL (the pH indicator changes color as a function of pH) and (2) a percentage of acid between 30 and 85% for a mixture of acid and base. The pH indicator is diluted to a total volume of 40 μL with water, while the total acid/base mixture volume is set to 200 μL.

The following code blocks illustrate how to apply ProcessOptimizer to this optimization problem and assume that ProcessOptimizer has been installed in a Python environment.^10^

The first step involves importing the needed Python package and defining the search space, which in this case includes the amount of indicator and the percentage of acid in the mixed buffer. This is done using the following Python code:
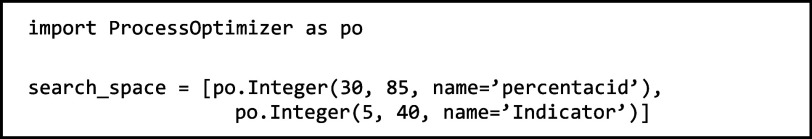
Next, we instantiate the optimizer and specify the number of initial points for the optimization process:
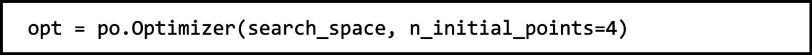
With the setup complete, we can now ask the optimizer for the parameters for the next experiment:
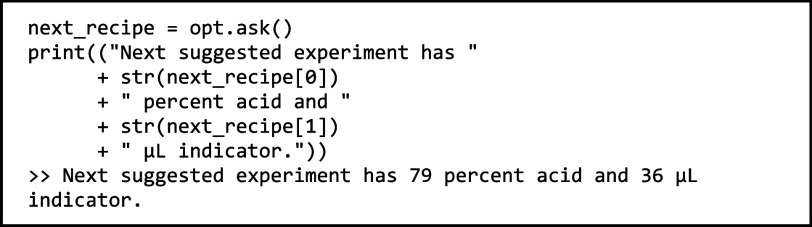
The value of the next_experiment variable instructs the user to perform an experiment with 79% acidic content of the pH buffer and 36 μL of pH indicator.

We then simulate conducting the experiment. To do this, we import color_pH model system and run its get_score() method with the suggested experiment parameters as input:
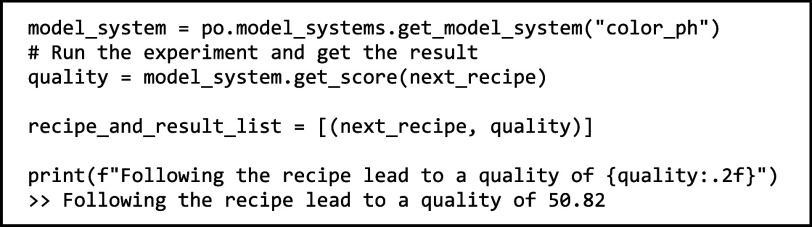
After simulating the experiment and obtaining the result, we return this result to the optimizer:
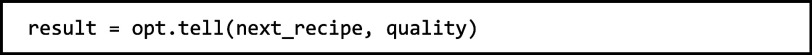
The opt.ask() and opt.tell() commands can be repeated until a recipe that satisfies the quality target of the user has been found.

The code above made use of a limited number of variables/settings:

- “search_space” is a list of dimensions that constitutes the overall design space in which the user is searching for a minimum. Dimensions can be “Real” (decimal numbers), “Integer” (integers), or “Categorical” (list of possible selectors, such as “EtOH”, “MeOH”, “*i*PrOH”). Numeric dimensions are given as (“min_value”, “max_value”, [optional]“Name”).
- “n_initial_points” is the number of experiments that will be suggested with no consideration of existing experimental data. These initial experiments are, by default, chosen via Latin hypercube sampling (LHS) to give a good coverage of each parameter in the search_space.
- “next_recipe” is a transient variable holding the recipe (formatted as a vector) for the next experiment.
- “opt” is the instantiation of the entire optimization. In the example above, the optimizer is instantiated with only knowledge of the design space and the intention of performing four initial experiments before the first regression model is fitted. However, in this, a number of options are given to allow the user to use different machine learning models for regression, different acquisition functions to help suggest the next experiment, knowledge of the design space and possible transformations (e.g., normalization) of the features, and options for multiobjective optimization or handling of modeled experimental noise. The rest is given by carefully chosen preset default values.
- “result” is an object containing the data acquired during the optimization as well as all the interim regression models. “result” is used for plotting of the regression model and for inquiries to the models’ expected minimum (both estimated value and estimated standard deviation at the expected optimal recipe). “result” is the returned value after having performed a “tell” command to the optimizer; it can also be produced as a trait of the optimizer (“result = opt.get_result()”)

## Methods and Materials

**Figure 2:**
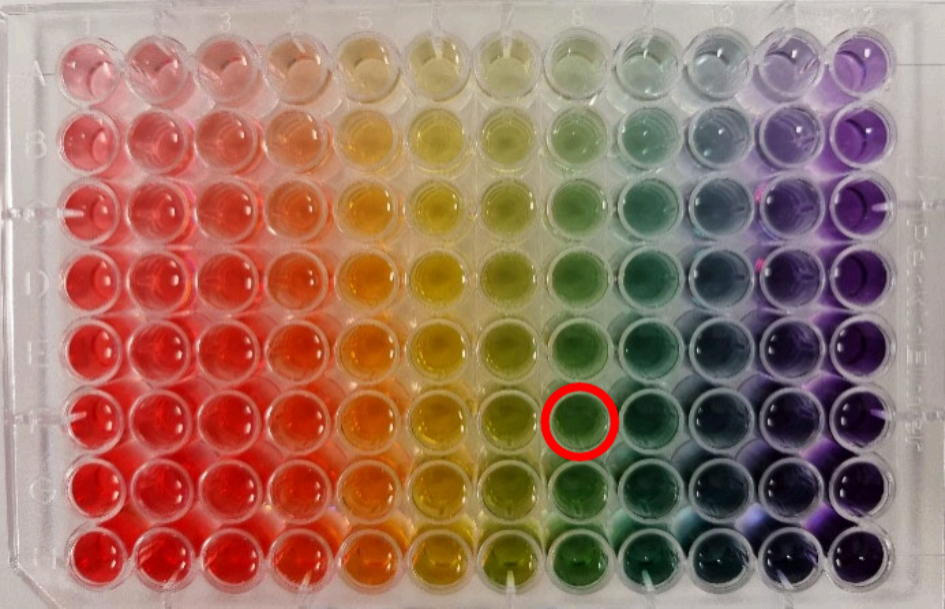
Possible outcomes of mixing a universal pH indicator with known and controlled amounts of acidic and basic buffers as described in the Supporting Information. The color that is the target of the optimization is circled in red. The full corpus of the data is assumed to be unavailable to the algorithm and the scientist.

To simulate an experiment in the lab reproducibly and transparently, we prepared different combinations of concentration of universal pH indicator and balance of acid and base in a 96-well SBS plate (see Figure 2). We then established the color of each well in the L*a*b color space and established this as a benchmarking data set (ModelSystem) in the ProcessOptimizer repository, which allows the user to quickly and effortlessly simulate making repeated physical experiments. The concrete objective is given by minimizing the calculated distance between an observed color and a target color. There are many metrics for distances in color spaces; here Δ*E* is a calculated difference in sensation of colors.^9^

Like most other optimization packages, ProcessOptimizer assumes that the user is working on a minimization problem and will suggest new experiments to this end. In the case presented here, the distance to the desired color is naturally a minimization process.

All code in this paper was run on a computer with Microsoft Windows 10 Enterprise (version 10.0.19045 Build 19045) and Python 3.9.1 installed. A new virtual Python environment was created, and ProcessOptimizer version 1.0.0 was installed with the command “pip install ProcessOptimizer”. The full list of installed packages is available in Supporting Information S4. All code in this paper was run in this environment in the order it appears in the paper, and the return is what is reported here. The full example can be found in a Jupyter Notebook in the following repository: https://github.com/novonordisk-research/ProcessOptimizer/blob/6c85018db95a79fbd479551270474089add3bb2b/examples/color_pH.ipynb.

## Results

To highlight the ability of ProcessOptimizer to optimize a physical process, we present a colorimetric pH adjustment. Using an easily prepared wide-range buffer series and a universal pH indicator, a wide range of colors and color saturations can be created experimentally (Figure 2).

To exemplify a scientist’s search for an optimal recipe in the presence of many plausible solutions, we show the algorithm’s approach to finding the amount of indicator and acid-to-base ratio to produce a specific color. In this experiment, we are searching for a mixture that results in a green color (red circle in Figure 2).

The obtained color is compared to a desired color by calculating the Δ*E* value of the *L*, *a*, *b*-encoded observed colors. The difference between the observed color for a given recipe and the desired color, Δ*E*, is iteratively fed back to the algorithm in a “Design–Make–Test–Evaluate” loop. In the current example, all data are pregenerated, but the optimization will be searching for a solution from scratch, thereby mimicking a real-world case in which the scientist does not know the best recipe for the task at hand.

Table 1 shows the experiments and the progression of the iterative model building process. The first four entries (Table 1, entries 1–4) constitute the results of recipes that are given by the algorithm to form an initial body of data from which to make a first model. The result of each experiment is given to the model as “result = opt.tell([80, 35], 50.8)” (example from entry 1). Entries 5–8 are iteratively suggested by the algorithm (by using the “opt.ask()” command). After eight experiments, the optimized recipe is extracted (Table 1, “Result”) by “po.expected_min(result)”. The results show that the optimized recipe results in the optimal color (confirmed by a calculated Δ*E* of 0).

**Table 1:**
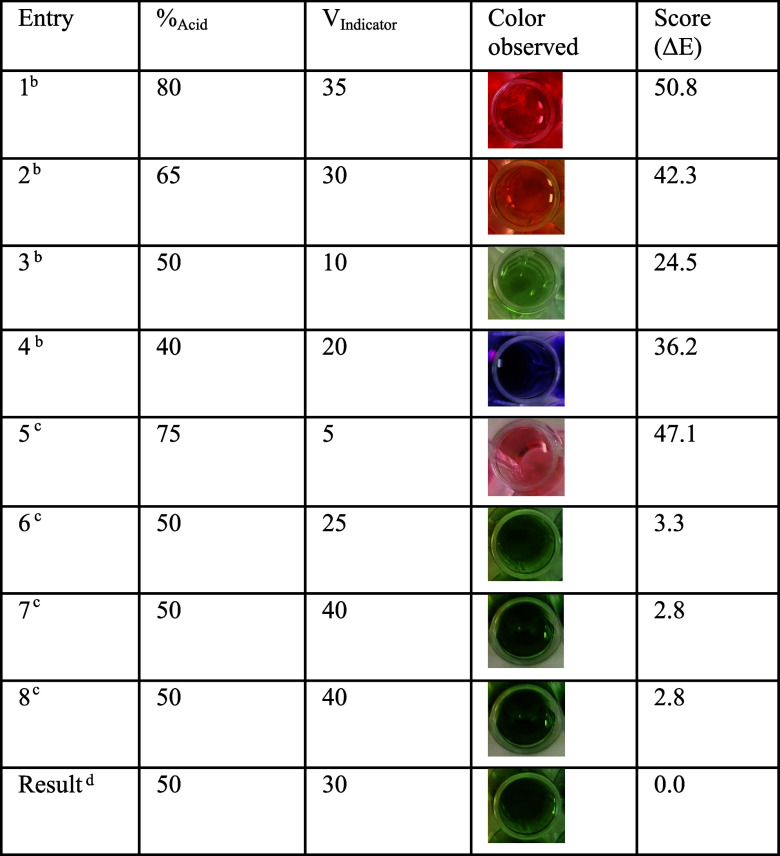
Progression of Iterative Optimization in the Search for a Recipe That Will Produce a Green-Colored Liquid^a^

a Recipes suggested by ProcessOptimizer are rounded to the nearest multiple of 5, and data are extracted from the preacquired experimental data base. Colors are numerically scored using the calculated distance to the desired green color (Δ*E*). See the Supporting Information for details.

b Designed as part of the four initial LHS experiments.

c Experiment designed by the Bayesian optimization learning loop.

d The expected best recipe from a model trained on 8 data points is extracted and performed.

The optimized model can be plotted with built-in features of ProcessOptimizer. As such, the multidimensional regression model is plotted in Supporting Information S3 as a result of the command “po.plot_objective(result)”.

Through this example, it is evident that the ProcessOptimizer package allows for a streamlined, efficient, and user-friendly approach to process optimization. By simplifying the setup and execution of the optimization process, it enables scientists to focus on the results and their implications rather than the intricacies of the optimization algorithms.

## Key Features of ProcessOptimizer

ProcessOptimizer offers a range of features that make it a user-friendly and versatile tool for real-world process optimization, usable out of the box:

(1) Comprehensive working examples of the most important features to get started (see: https://github.com/novonordisk-research/ProcessOptimizer/blob/6c85018db95a79fbd479551270474089add3bb2b/examples/walkthrough/readme.md).
(2) Comprehensive Plotting Features: The package includes several plotting features to visualize the underlying regression model and help users determine whether the optimization is complete (see Supporting Information S3).
(3) Works with a combination of continuous factors (decimal numbers), discrete numerical factors (integer numbers), and categorical factors (e.g., solvents, colors, suppliers).
(4) Constraints in the design space, including mixture designs and min, max, or specific values for individual factors.
(5) Batch-Mode Operation: ProcessOptimizer can provide multiple experiment suggestions at once, enhancing efficiency and productivity, when practical circumstances favor executing multiple runs at a time.
(6) Multiobjective Bayesian Optimization: The package supports Pareto optimization using NSGA-II, enabling multiobjective Bayesian optimization.
(7) Benchmarking and Noise Management: A built-in ModelSystem class allows users of ProcessOptimizer to easily run benchmarks.
(8) Model Uncertainty: ProcessOptimizer allows users to easily manage experimental noise modeling by adding or removing it from representations such as plots.

At present, ProcessOptimizer does not support design spaces that only contain categorical factors. Multiobjective optimization with ProcessOptimizer does not support constraints. Furthermore, investigations are ongoing regarding adding features such as initial DoE-inspired experimental design and multifidelity optimization. Implementation of new features is driven by identified user needs while maintaining a focus on user friendliness and usability within real-world experiments.

## State of the Art

Many other Python packages also offer access to Bayesian optimization, including both packages tailored to specific branches of science and more generic packages.

To assist the reader in getting an overview of the state of the art in the field, we have assessed a number of current versions of these packages on recency, availability of easy-to-follow examples, plotting capabilities, and a number of features of high importance while optimizing physical experiments. The resulting curated list is presented in Supporting Information S1. As can be seen from the table, there are a number of relevant packages which are currently being maintained. The ultimate choice of package should depend on the use case, experience of the user, and subjective preferences.

The aim of the development of ProcessOptimizer is to provide a user-friendly and robust experience for new practitioners within a wide range of experimental fields/sciences. As such, it does not offer cutting-edge mathematics, e.g., acquisition functions, as this is a fast-moving subject with active discussion about the optimal approach.^11^

## Conclusion

ProcessOptimizer is a powerful, user-friendly Python package that brings advanced Bayesian optimization techniques to experimental scientists and professionals in various fields. With its unique features and easy-to-use interface, it can be readily used by scientists with just a minimum of training in Python.

In addition, it also offers benchmarking, noise management, multiobjective Bayesian optimization, batch-mode operation, and comprehensive plotting features.

## Data Availability

ProcessOptimizer is publicly available free of charge at https://github.com/novonordisk-research/ProcessOptimizer. It can be installed using the standard package-management system in Python. Data presented in the present paper are available as part of the repo.

## Supporting Information Available

S1: survey of open-source Python packages for Bayesian optimization; S2: experimental setup and analysis; S3: code examples for the example presented in the current paper, including additional plotting capabilities found in the ProcessOptimizer package; S4: list of installed packages in the install of PO used for the examples (PDF)
