# Supplementary material for: ProcessOptimizer, an Open-Source Python Package for Easy Optimization of Real-World Processes Using Bayesian Optimization: Showcase of Features and Example of Use
Source: J Chem Inf Model. 2025 Feb 7;65(4):1702–7. doi: 10.1021/acs.jcim.4c02240 (PMC11863379; doi:10.1021/acs.jcim.4c02240)
Supplement: Supplementary file 1 — ci4c02240_si_001.pdf [file ci4c02240_si_001.pdf]

ProcessOptimizer, an open-source python package for easy optimization of real world processes using Bayesian optimization. Show-case of features and example of use

Søren Bertelsen[1*], Sigurd Carlsen[4], Søren Furbo[1], Morten Bormann Nielsen[3], Aksel Obdrup[5], Rolf Taaning[2]

[1]Department of Automation and Process Optimisation, Digital Science and Innovation, Novo Nordisk A/S, Måløv 2760, Denmark.
[2]Department of Late-Stage Chemical Development, Chemistry Manufacture and Control, Novo Nordisk A/S, Bagsværd 2880, Denmark.
[3]Danish Technological Institute, Kongsvang Allé 29, DK-8000 Aarhus C, Denmark.
[4]Teal Medical, Copenhagen 2300, Denmark.
[5]Department of Digitalization, Global IT & Digital, Topsoe A/S, Kgs. Lyngby 2800, Denmark.

Corresponding author: sqbl@novonordisk.com

Content:

S1: A survey of open-source python packages for Bayesian optimization
S2: Experimental setup and analysis
S3: Code examples for example presented in current paper including additional plotting capabilities found in the ProcessOptimizer package
S4: List of installed packages in the install of PO use for the examples

S1: A survey of open-source python packages for Bayesian optimization

We surveyed the available open-source python packages that supports Bayesian optimization and scored them according to capabilities, usability, and other measures. Survey was performed 2025-01-13. During our selection of packages to evaluate, the following criteria were considered:

- The ability to install the package from pypi or conda.
- Support for physical experimentation, including decoupling getting suggestions for the next experiment and providing the results.

Package (repo, reference)	Date of latest pypi release [version]	"getting started" code example(s) for full optimization loop available (link)	Supports multi-objective optimization (algorithm)	Plotting capabilities	Supports constraints in input space	Batch operation, getting more suggested parameter sets at the same time
BayesianOptimization (https://github.com/bayesian-optimization/BayesianOptimization)	2024-07-10, [2.0.3]	✓	✗	✗	✓	✗
ScikitOptimize<br><br>(https://github.com/holgern/scikit-optimize)	2024-06-04,<br><br>[0.10.2]	✓	✗	✓	✓	✓
Botorch and Ax<br><br>(https://github.com/pytorch/botorch, https://github.com/facebook/Ax)<br><br>In the opinion of the authors, this combination offers a rich and deep Bayesian Optimisation development platform, but not one that is easy to use for non-programmers.	Botorch: 2024-09-17 [0.12.0]<br><br>Ax: 2024-09-23 [0.4.3]	✓	✓	✓	✓	✓
Optuna<br><br>(https://github.com/optuna/optuna)	2024-11-12 [4.1.0]	✓	✓	✓	✓	✓
Summit<br><br>(https://github.com/sustainable-processes/summit)	2023-02-14 [0.8.9]	✓	✓	✓	✓	✓
Phoenics<br><br>(https://github.com/aspuru-guzik-group/phoenics)	2020-05-22 [0.2.0]	✓	✓	✗	✗	✓
SafeOpt<br><br>(https://github.com/befelix/SafeOpt)	2020-04-16 [0.16]	✓	✗	✓	✗	✗
Edbo<br><br>(https://github.com/b-shields/edbo)	2020-11-18 [0.1.0]	✗	✗	✓	✗	✓
Trieste<br><br>(https://github.com/secondmind-labs/trieste)	2024-11-06 [4.2.2]	✓	✓	✓	✓	✓
Nextorch<br><br>(https://github.com/VlachosGroup/nextorch)	2023-12-12<br><br>[0.4.0]	✓	✓	✓	✗	✓

Bayes-skopt/bask<br><br>(https://github.com/kiudee/bayes-skopt)	2023-07-19,<br><br>[0.10.9]	✓	✗	✗	✗	✓
Gryffin<br><br>(https://github.com/aspuru-guzik-group/gryffin)	2022-08-03,<br><br>[1.0.0]	✓	✓	✗	✓	✓
GPyOpt<br><br>(https://github.com/SheffieldML/GPyOpt)	2020-03-19<br><br>[1.2.6]	✗	✗	✓	✗	✓
SMAC3<br><br>(https://github.com/automl/SMAC3)	2023-07-24<br><br>[2.2.0]	✓	✓	✓	✗	✗
BayBE[A]<br><br>(https://github.com/emdgroup/baybe)	2024-11-06,<br><br>[0.11.3]	✓	✓<br>(through guided scaling/weighing of multible readouts)	✓	✓	✓
Dragonfly<br><br>(https://github.com/dragonfly/dragonfly)	2022-10-02,<br><br>[0.1.7]	✓	✓	✗	✗	✓
GAUCHE<br><br>(https://leojklarner.github.io/gauche/)	2023-12-11,<br><br>[0.1.6]	✓	✗	✗	✗	✗

(Github search for "Bayesian optimization" returns 1.8k repositories) [A]BayBE seems particularly suited for applications in chemistry with custom domain specific encodings for chemical substances.

S2: Experimental setup and analysis

Experimental setup:

Acidic and basic buffers were made in accordance with literature procedure. (Carmody, W. R. Easily prepared wide range buffer series. Journal of Chemical Education 1961, 38 (11), 559. DOI: 10.1021/ed038p559.)

Acidic buffer: 0.20 M Boric acid, 0.050 M Citric acid.

Basic buffer: 0.10 M Na_3PO_4

Commercially available universal pH indicator was used. (https://www.sigmaaldrich.com/DK/en/product/mm/109175)

Using a d^2 robotic dispenser from UK Robotics (https://ukrobotics.com/shop/d2-dispenser/), a known amount of indicator is dispensed into a 96 well flat bottomed, transparent SBS plate. For each row, the volume of indicator is increased by 5 µL, in a way so that all wells in row “A” receive 5 µL of indicator, all wells in row “B” receive 10 µL and all wells in row “H” receive 40 µL of indicator. Demineralized water is added to necessary wells to give an intermittent total volume of 40 µL.

To each column, known amounts of acidic buffer and basic buffer is added. To column “1” is added 170 µL acidic buffer and 30 µL basic buffer (=85 % acid). To column “2” is added 160 µL acidic and 40 µL basic buffer (=80 % acid). Accordingly, to column “12” is added 60 µL acidic buffer and 140 µL basic buffer (=30 % acid).

Image of the resulting colors was recorded by photography with constant white balance across the image. All L*A*B colors are extracted from a single image using OpenCV.

Full overview can be found in https://github.com/novonordisk-research/ProcessOptimizer/blob/6c85018db95a79fbd479551270474089add3bb2b/ProcessOptimizer/model_systems/data/color_pH_data.csv.

S3: Code examples for example presented in current paper including additional plotting capabilities found in the ProcessOptimizer package

Optimizing a chemical reaction

As a very simple example of optimizing a chemical reaction, we try to find the mix of universal indicator, acid and base that gives the best green color. While this is a very simple experiment, it shows the general approach one would follow in optimizing a chemical reaction according to yield or purity.

We have made a full factorial mix of different levels of indicator while varying the percentage of acid (`V(acid)/(V(acid) + V(base))*100 %`), and measured the resulting color:

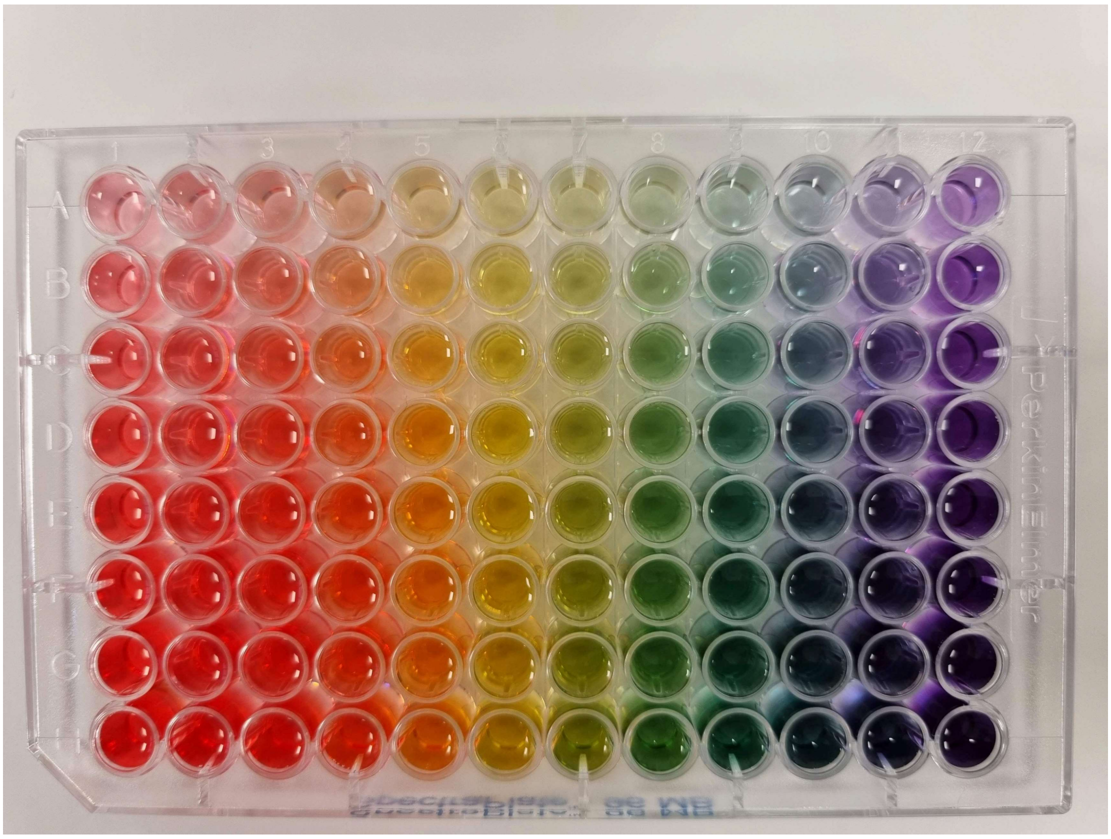

When in the lab, you would normally do one experiment at a time. To simulate this, we have made it possible to look up the result from any recipe. In other words, we can do theoretical experiments that mimic what you would see if you were to do laboratory experiments one at a time. This behavior is captured by the model system color_pH.

We first set up the parameter space:

In [39]:

```
import ProcessOptimizer as po

search_space = [
       po.Integer(30,85, name="pct_acid"),
       po.Integer(5,40, name="vol_indicator")
       ]
```

We can then set up the processOptimizer:

In [40]:
```
opt = po.Optimizer(search_space, n_initial_points=4)
```

We can now ask the optimizer to suggest the next recipe to try, and use the model system to simulate following the recipe and telling us how good the result is:

In [41]:
```
# Get the first suggested experiment
next_recipe = opt.ask()
print(("Next suggested experiment has "
      + str(next_recipe[0])
      + " percent acid and "
      + str(next_recipe[1])
      + " µL indicator."))
```

```
Next suggested experiment has 79 percent acid and 36 µL indicator.
```

Using the model system separately

The model system has a method called `get_score()` which simulates following the recipe, measuring the resulting color, and evaluating how close it is to the wanted color.

In a lab setting, you would run the experiment instead, and record the results.

We also start saving the recipes simulated and their resulting qualities in the parameter `recipe_and_resuilt_list`.

In [42]:
```
model_system = po.model_systems.get_model_system("color_pH")
# Run the experiment and get the result
quality = model_system.get_score(next_recipe)

recipe_and_result_list = [(next_recipe, quality)]

print(f"Following the recipe lead to a quality of {quality:.2f}")
```

```
Following the recipe lead to a quality of 50.82
```

Now, we can tell the optimizer about the recipe we just "followed".

In [43]:
```
result = opt.tell(next_recipe, quality)
```

We can keep doing this for a few runs.

In [44]:
```
for i in range(7):
    next_recipe = opt.ask()
    quality = model_system.get_score(next_recipe)
    recipe_and_result_list.append((next_recipe, quality))
    result = opt.tell(next_recipe, quality)
```

Expected minimum

We can now find the expected minimum of the model:

In [45]:
```
expected_minimum = po.expected_minimum(result)
print(
    f"The expected minimum is {expected_minimum[0][0]} percent acid and "
```

```
    f"{expected_minimum[0][1]} µL indicator. It has an expected quality of "
    f"{expected_minimum[1]}."
)
```

```
The expected minimum is 49 percent acid and 31 µL indicator. It has an expected q
uality of -0.4387244862982769.
```

The true minimum is at 50 percent acid and 30 µL indicator, but since the experimental mixtures are only made at whole multiples of 5 percent acid and 5 µL indicator, 49 percent acid and 31 µL indicator gives the same quality.

The experimental quality at the true minimum is 0 by definition, so the model estimates a bit better quality at the minimum.

Table with overview of results

We can now make a table with an overview of the tested recipes and their results. Rember, lower score is better:

In [46]:
```
for recipe, quality in recipe_and_result_list:
    print({
        "pct_acid": recipe[0],
        "vol_indicator": recipe[1],
        "score": round(quality,1)
    })
```

```
{'pct_acid': 79, 'vol_indicator': 36, 'score': 50.8}
{'pct_acid': 65, 'vol_indicator': 27, 'score': 42.3}
{'pct_acid': 51, 'vol_indicator': 9, 'score': 24.5}
{'pct_acid': 37, 'vol_indicator': 18, 'score': 36.2}
{'pct_acid': 74, 'vol_indicator': 5, 'score': 47.1}
{'pct_acid': 50, 'vol_indicator': 23, 'score': 3.3}
{'pct_acid': 49, 'vol_indicator': 40, 'score': 2.8}
{'pct_acid': 51, 'vol_indicator': 38, 'score': 2.8}
```

Plotting

Finally, we can plot the result object to inspect the model.

Plot Objective in n-dimensions

In [47]:
```
# Make additional plot with measurement uncertainty

po.plot_objective(result,
                  pars = 'expected_minimum',
                  plot_options = {"interpolation": "bicubic"},
                  );
```

Figure

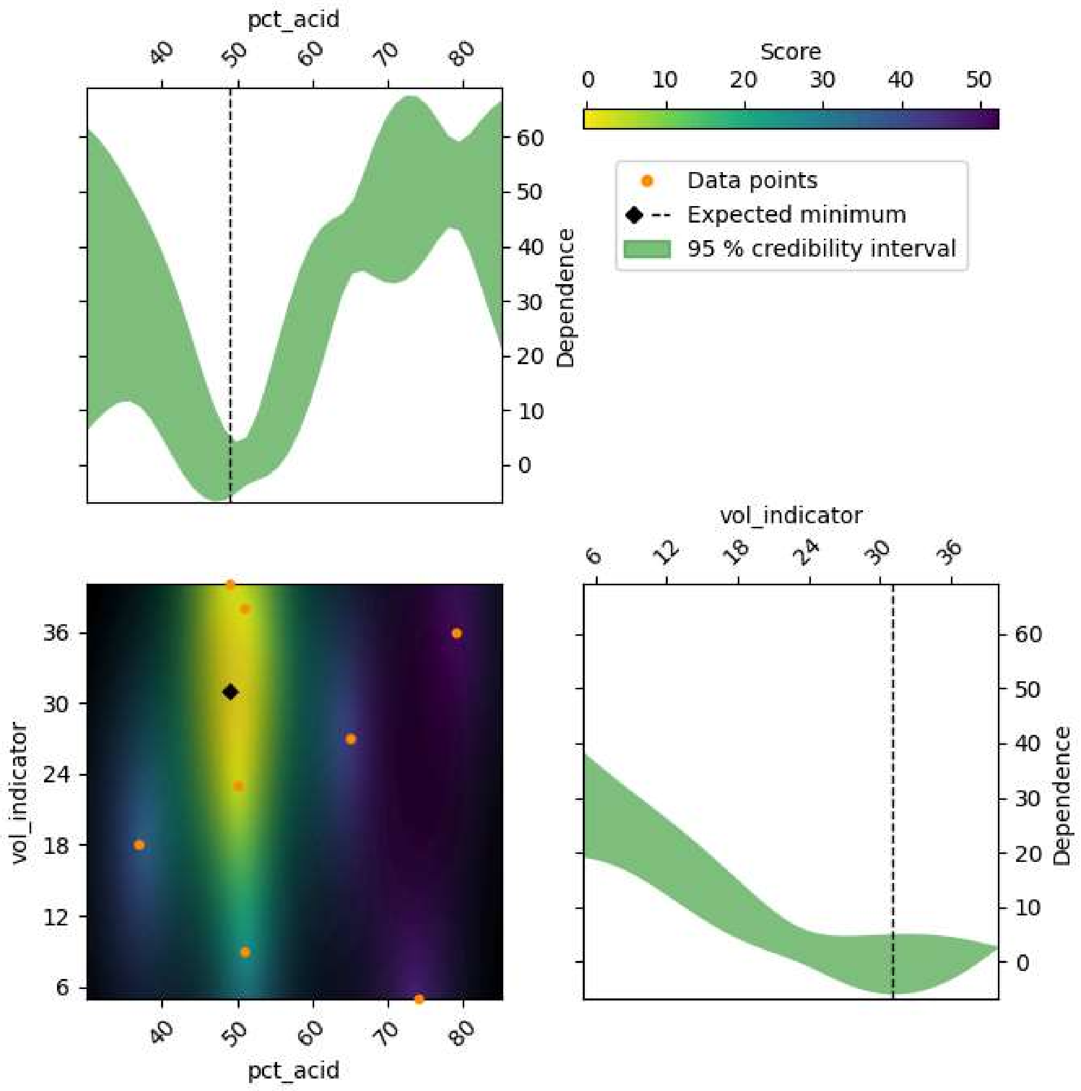

The 1D plots on the diagonal shows the credible interval of the objective function when one control parameter is changed. The colors of the 2D plot in the lower left shows the expected value of the objective function when two control parameters are changed. In this example, there are only two control parameters, so that is the entire model. In models with more control parameters, the dependency on each pair of variables would be shown as separate 2D plots, all situated in the lower left triangular part of the plot. This plotting type scales to any number of control parameters, including real, integer and categorical parameters.

Through keywords and settings in `plot_options`, this plot can show (or hide) the uncertainty of the model in both 1D graphs (the diagonal line of subplots) as well as 2D graphs (subplots in the lower left triagonal part of the plot). Be mindfull that all graphs are reductions of dimensionality and hence, represent a "slice" of the full picture with all other control features kept constant.

Furthermore, any slice of 1D and 2D plots can be called in situation where the experimenter might be interested in locking a number of control parameter and choosing the optimal setting of the other control parameters: this is done through the `pars` keyword.

Plot objective in one dimension

In [48]:
```
po.plots.plot_objective_1d(result,
                           pars = 'expected_minimum');
```

Figure

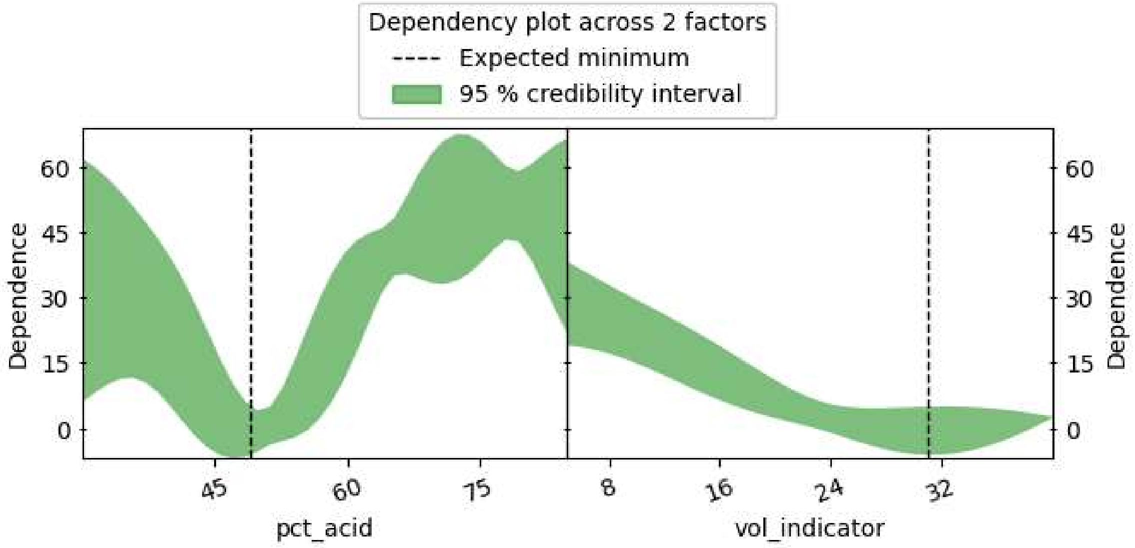

A simpler, more clean plotting of the models expected outcome. Now, only 1D plots are shown. This is expected to highlight the importance of individual control features even further.

Plot Evaluations

In [49]:
```
po.plot_evaluations(result);
```

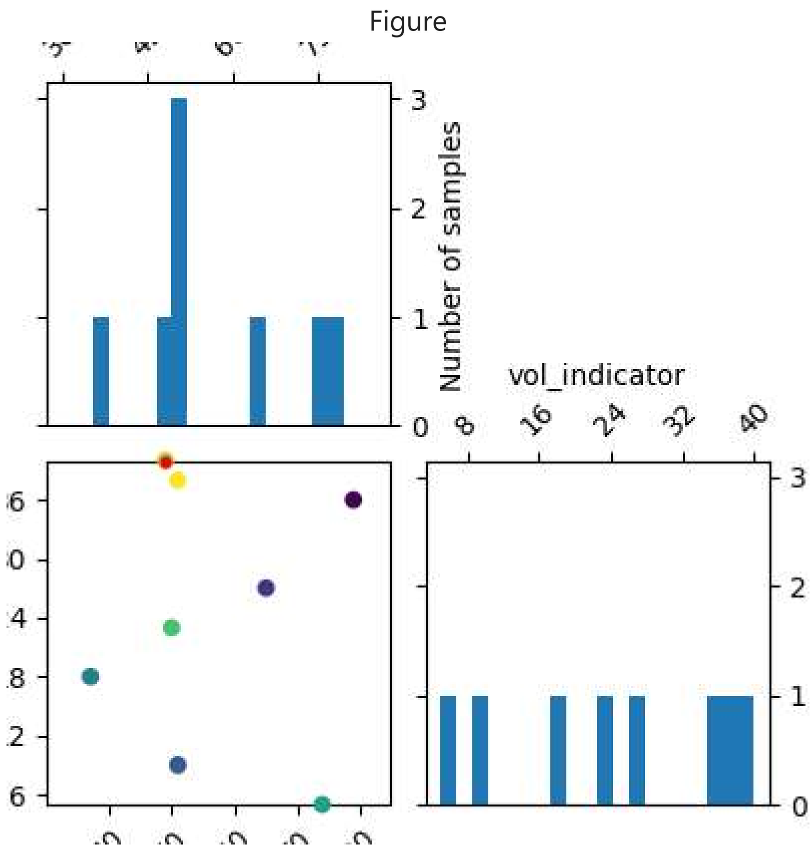

Plot evaluations follows the same layout and structure as plot_objective (see above). It can be used as a fast visual check on whether the full control feature space has been covered by experiments. (Remember that projections of high dimensionality into 1D or 2D might hide complexity). The coloring of the dots in the 2D-plotting area show the sequence of experiments from dark blue (first experiment) through green to yellow. Latest experiment is marked in red.

Plot Convergence

In [50]:
```
po.plot_convergence(result);
```

The convergence plot shows the best obtained result as a function of iteration. It is expected that the best observed result does not move at every iteration due to the neccesary explore/exploit trade-off when using Bayesian Optimization. Remember that the convergence plot will only show the best observed result, so a constant `min(obtained_result)` might reflect creating a more accurate model, that can in turn allow the models to predict a recipy with even better results.

Plot Regret

In [51]:
```
po.plots.plot_regret(result);
```

A cumulative regret plot will show the accumulated cost of not having run at the best settings from the start. Optimally seen, the accumulated cost should be as low as possible when the experimenter reaches a set of acceptable conditions for the process being optimized. The plot is very useful when comparing several optimization strategies.

Experimental plots

In [52]:
```
po.plot_expected_minimum_convergence(result, sigma=0.2);
```

Figure

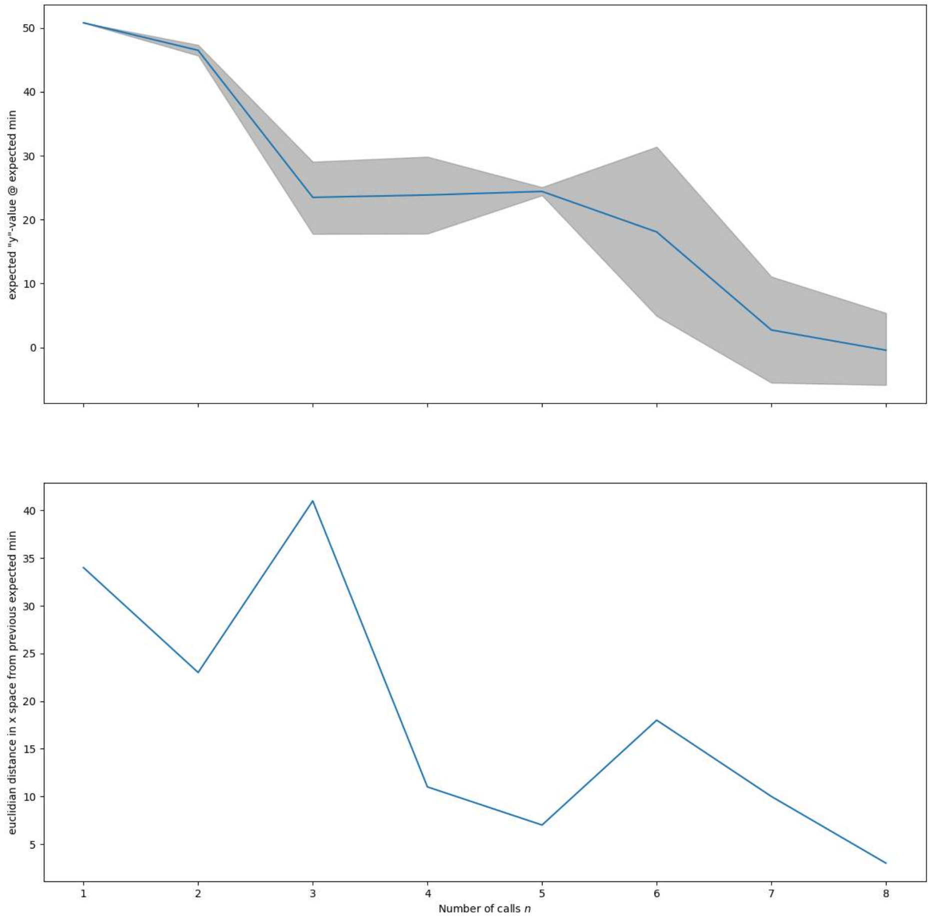

The plot_expected_minimum_convergence plot (above) is a more exploratory plot. It is meant to help the user judge the progression of the optimization. The upper panel shows the expected value (incl model uncertainty) that can be reached by the process given the current model (as a function of interation number). The value should hopefully converge to a smaller number, while the model uncertainty should decrease as a signal for a more data covered and completed model. The lower panel shows the euclidian distance in the design-space of the experiments between the expected minimum at the current iteration compared to the previous iteration. In a case in which all factors have

importance for the process, the lower panel should converge as a sign that the model converges towards a single perceived optimum.

```
In [53]: po.y_coverage(result, return_plot=True);
```

Figure

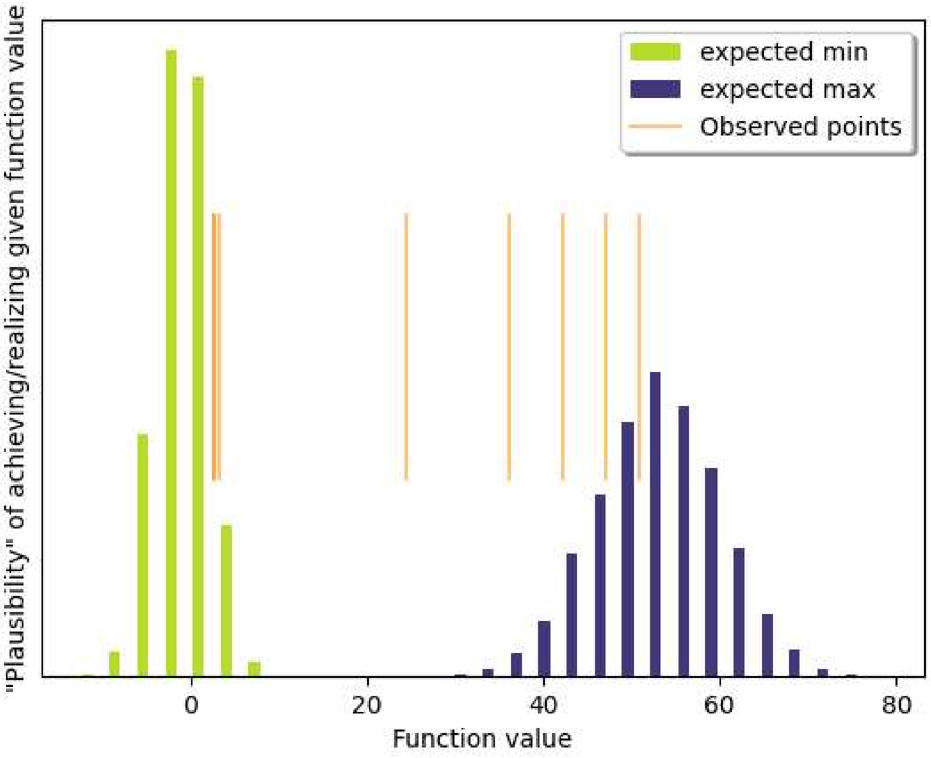

The plot y_coverage is another exploratory plot meant to convey an understanding of the model uncertainty and the underlaying data. Orange bars show the function values that has been told to the model, while two histograms show the current models uncertainty in the expected maximum and minimum values. Signs of a good model would be that the interesting extremum (the minimum in our case) is more data covered and has less uncertanty compared to the other extremum. Personally, I would tend to trust a model that has learned from data that has realised values close to the objective (a good minimum in our case).

SRFU 2024-08-16: A fully data covered model would have the same uncertainty for the two extrema, right? And would also be a good model.

Additional plots

To demonstrate the plotting capabilities for multiobjective optimization, we can imagine a scenario in which the color indicator has a price that is a limit to out productivity (again, this is just an example to demonstrate plotting functionality). In doing so, we start a new optimization in which we highlight that we have two objectives, and when we tell the results of a experiment, we now have to enter a list of measurements containing the score of the color as well as the cost of the color. As we are trying to learn two objectives

(and their connection) we expect to need a few additional experiments. In the code below, we perform 10 experiments.

In [54]:
```
opt_multiobjective = po.Optimizer(search_space, n_objectives=2, n_initial_points

recipe_and_result_list_multiobjective=[]

for i in range(10):
    next_recipe = opt_multiobjective.ask()
    quality_and_cost = [model_system.get_score(next_recipe),next_recipe[1]]  # c
    recipe_and_result_list_multiobjective.append((next_recipe, quality_and_cost)
    result_multiobjective = opt_multiobjective.tell(next_recipe, quality_and_cos
```

In [55]:
```
po.plot_objectives(result_multiobjective);
```

Figure

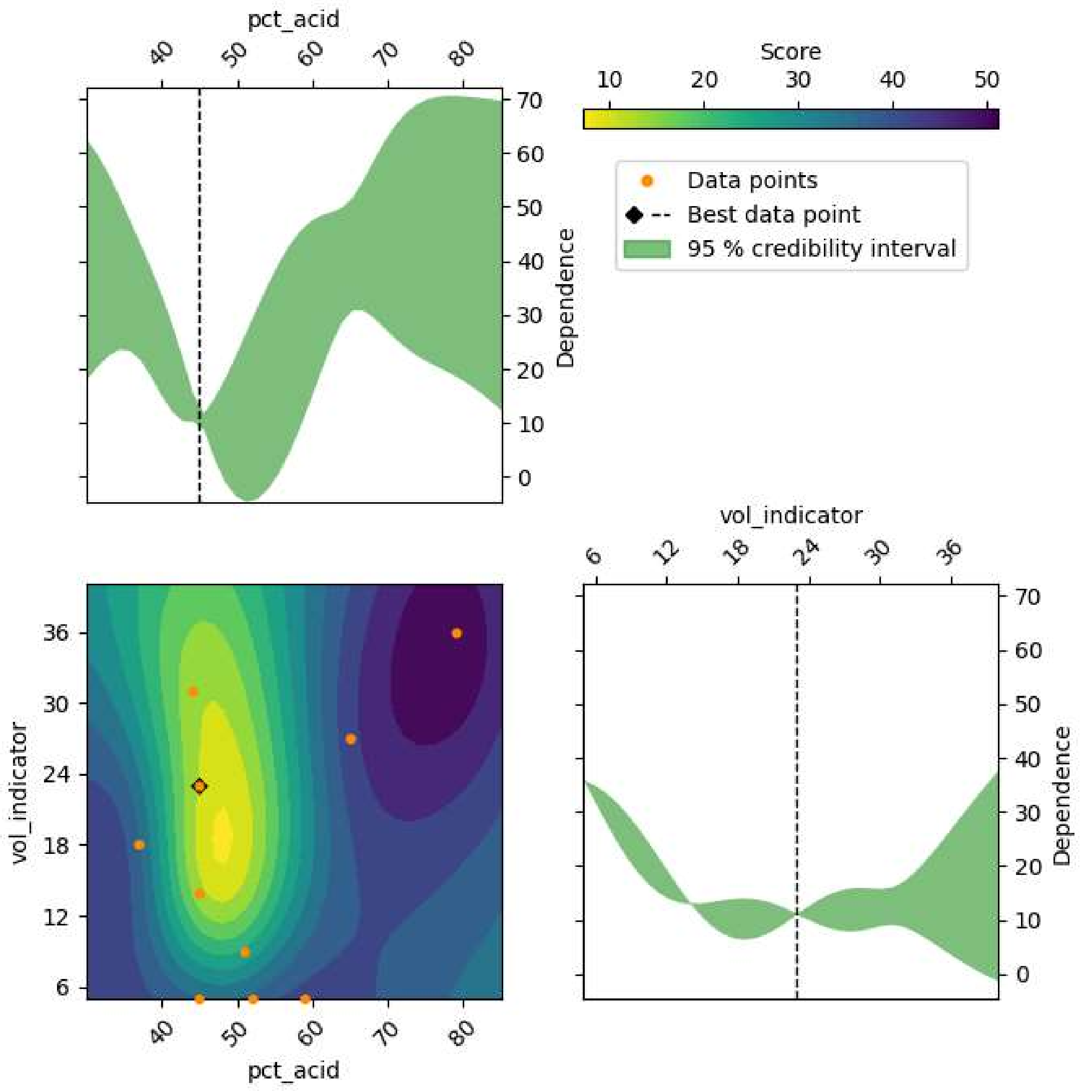

Figure

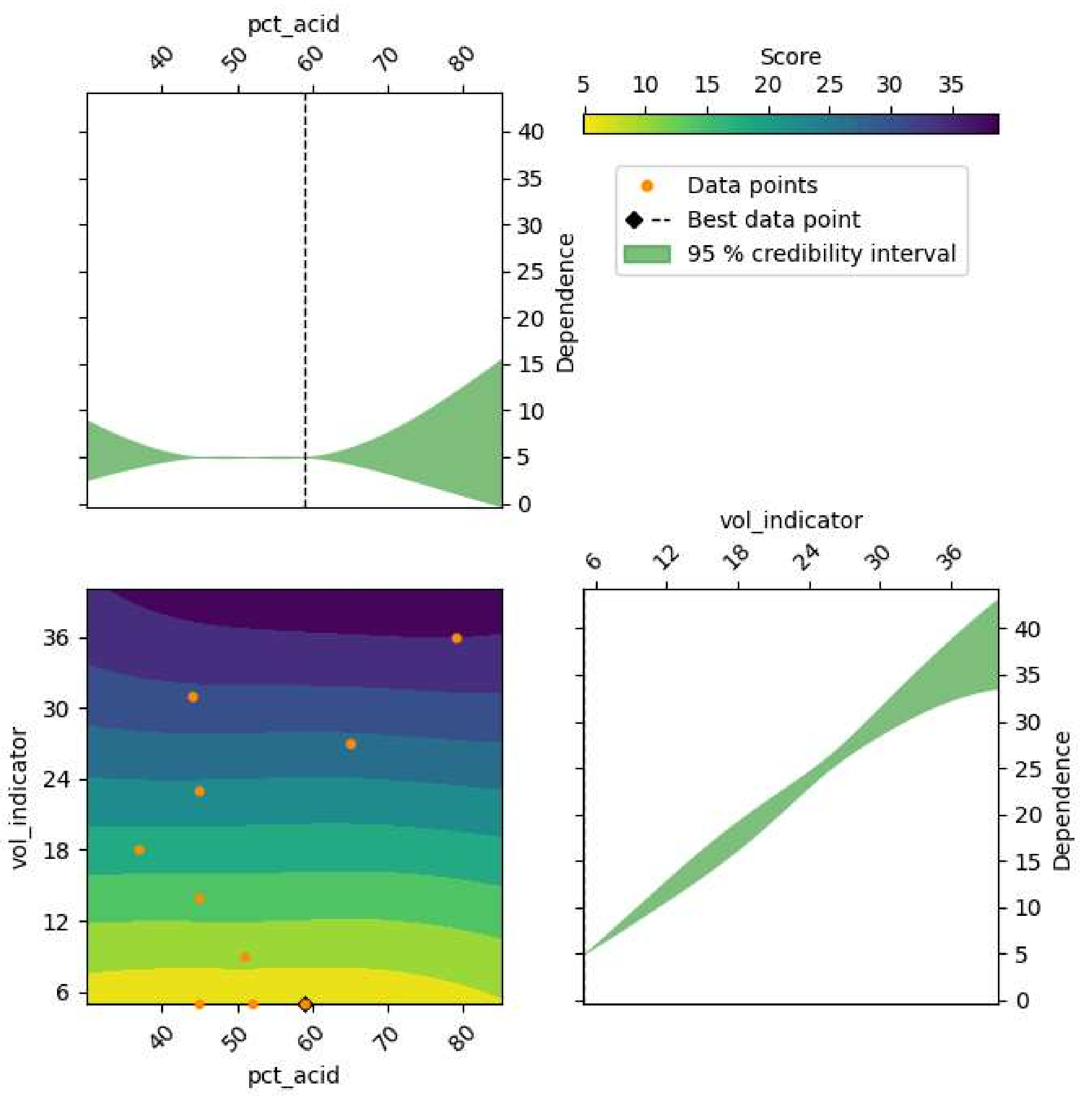

The first plot is a convenience functions that allows the user to plot the model for each objective defined in the experiment. As such plot_objectives() plots a list of plot_objective() plots (pay attention to the plural-s in the call of the function).

In [56]:
```
#ipympl is needed for the following cell to render. without ipympl, the user is
%matplotlib widget
po.plot_Pareto(opt_multiobjective, figsize=(8,8), objective_names=["Quality", "C
```

Figure

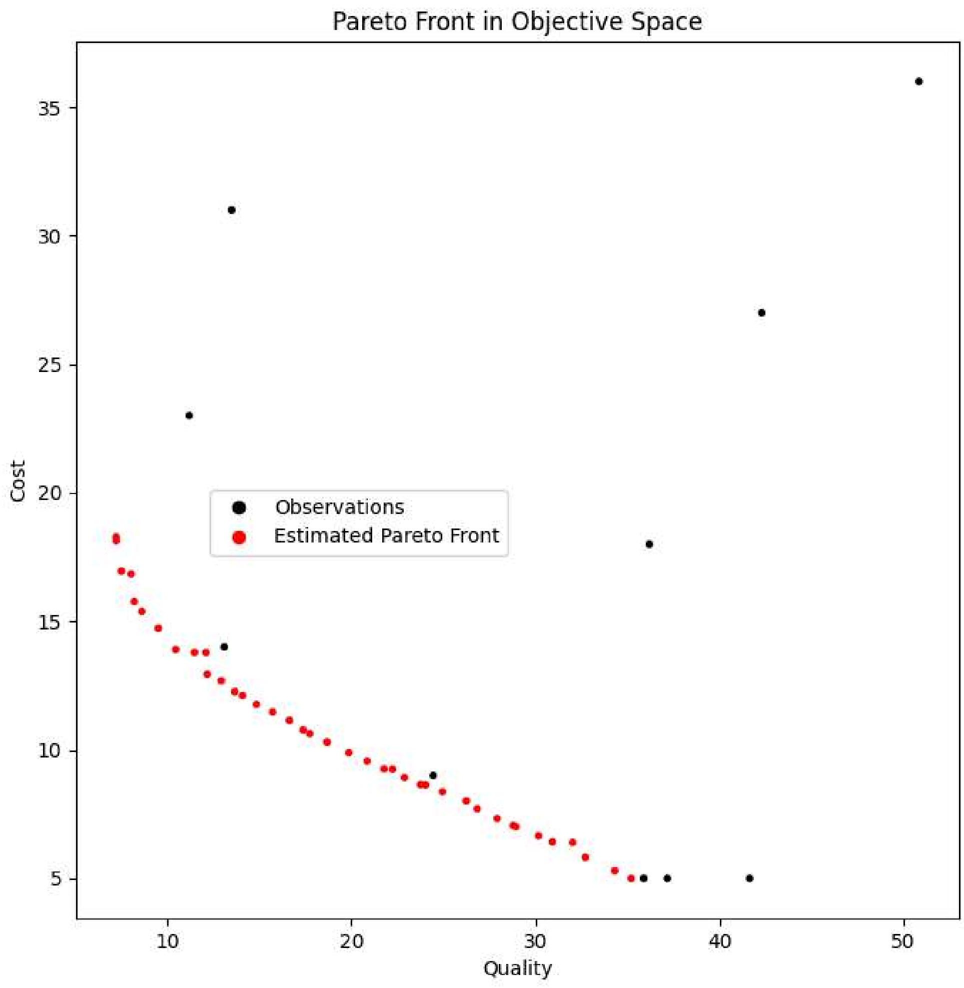

The Pareto front can easily be plotted and this plot will be interactive. As such, the user gets an overview of how the "quality" and the "cost" interact. Clearly, it is not possible to reach the intended green color without the use of indicator liqued (which is assumed to be the cost driver in this example). The black circles on the plot corresponds to realised, real experimental data. The red circles is the modelled Pareto-front of the process i.e. the points at which one objective cannot be further improved without being at the cost of a detrimental effect to the other objective(s). The user can hover the mouse over any point to see the suggested experimental recipe can will lead to that specific result (given model correctness).

Further plotting features of the Pareto front is included in the package.

```
In [ ]:
```

S4: List of installed packages in the install of PO use for the examples

Packages from installing PO

```
Package            Version
------------------- -----------
bokeh              3.4.3
contourpy          1.3.0
cycler             0.12.1
deap               1.4.1
fonttools          4.53.1
importlib-resources 6.4.5
jinja2             3.1.4
joblib             1.4.2
kiwisolver         1.4.7
MarkupSafe         2.1.5
matplotlib         3.9.2
numpy              2.0.2
packaging          24.1
pandas             2.2.2
pillow             10.4.0
pip                20.2.3
ProcessOptimizer   1.0.0
pyparsing          3.1.4
python-dateutil    2.9.0.post0
pytz               2024.2
PyYAML             6.0.2
scikit-learn       1.5.2
scipy              1.13.1
setuptools         49.2.1
six                1.16.0
threadpoolctl      3.5.0
tornado            6.4.1
tzdata             2024.1
xyzservices        2024.9.0
zipp               3.20.2
```

Packages necessary to run the example

```
Package            Version
------------------- -----------
asttokens          2.4.1
bokeh              3.4.3
colorama           0.4.6
comm               0.2.2
contourpy          1.3.0
cycler             0.12.1
deap               1.4.1
debugpy            1.8.5
decorator          5.1.1
exceptiongroup     1.2.2
executing          2.1.0
fonttools          4.53.1
importlib-metadata 8.5.0
importlib-resources 6.4.5
ipykernel          6.29.5
ipympl             0.9.4
ipython            8.18.1
ipython-genutils   0.2.0
ipywidgets         8.1.5
jedi               0.19.1
jinja2             3.1.4
joblib             1.4.2
jupyter-client     8.6.2
jupyter-core       5.7.2
jupyterlab-widgets 3.0.13
kiwisolver         1.4.7
MarkupSafe         2.1.5
matplotlib         3.9.2
```

matplotlib-inline	0.1.7
nest-asyncio	1.6.0
numpy	2.0.2
packaging	24.1
pandas	2.2.2
parso	0.8.4
pillow	10.4.0
pip	20.2.3
platformdirs	4.3.3
ProcessOptimizer	1.0.0
prompt-toolkit	3.0.47
psutil	6.0.0
pure-eval	0.2.3
pygments	2.18.0
pyparsing	3.1.4
python-dateutil	2.9.0.post0
pytz	2024.2
pywin32	306
PyYAML	6.0.2
pyzmq	26.2.0
scikit-learn	1.5.2
scipy	1.13.1
setuptools	49.2.1
six	1.16.0
stack-data	0.6.3
threadpoolctl	3.5.0
tornado	6.4.1
traitlets	5.14.3
typing-extensions	4.12.2
tzdata	2024.1
wcwidth	0.2.13
widgetsnbextension	4.0.13
xyzservices	2024.9.0
zipp	3.20.2
